# Use of Centre for Disease Control criteria to classify infections in critically ill patients: results from an interobserver agreement study

**DOI:** 10.1186/cc11715

**Published:** 2012-11-14

**Authors:** PMC Klein Klouwenberg, DSY Ong, LD Bos, FM de Beer, MA Huson, M Straat, LA van Vught, L Wieske, J Horn, MJ Schultz, T van der Poll, MJM Bonten, OL Cremer

**Affiliations:** 1 University Medical Centre, Utrecht, the Netherlands; 2 Academic Medical Centre, Amsterdam, the Netherlands

## Background

Correct classification of the source of infection is important in observational and interventional studies of sepsis. The Centre for Disease Control (CDC) criteria are most commonly used for this purpose, but the robustness of these definitions in critically ill patients is not known. We determined the interobserver agreement for classifying infections in the ICU.

## Methods

Data were collected as part of a prospective cohort of 1,214 critically ill patients admitted to two hospitals in the Netherlands between January 2011 and June 2011. Eight observers assessed a random sample of 168 out of 554 patients who had experienced at least one infectious episode in the ICU. Each patient was assessed by two randomly selected observers who independently scored the source of infection (by affected organ system or site), the plausibility of infection (rated as none, possible, probable, or definite), and the most likely causative pathogen. Assessments were based on a *post hoc *review of all available clinical, radiological and microbiological evidence. The observed diagnostic agreement for source of infection was classified as partial (that is, matching on organ system or site) or complete (that is, matching on specific diagnostic terms), for plausibility as partial (two-point scale) or complete (four-point scale), and for causative pathogens as an approximate or exact pathogen match. Interobserver agreement was expressed as percentage agreement and as a kappa statistic.

## Results

A total of 206 infectious episodes were observed in 168 patients. Agreement regarding the source of infection was 89% (183/206) and 69% (142/206) for partial and complete diagnostic agreement, respectively (Table 1). This resulted in an overall kappa of 0.85 (95% CI = 0.79 to 0.90). Agreement varied from 70 to 100% within major diagnostic subgroups. In the subgroup of 142 episodes with full diagnostic agreement on source of infection, the interobserver agreement for plausibility of infection was 83% and 65% on a two-point and four-point scale, respectively. For causative pathogen, agreement was 78% and 70% for an approximate and exact pathogen match, respectively.

**Table 1:** Agreement across the various sources of infection

Source of infection	Agreement/total number
Lower respiratory tract	86/90 (96%)
Community-acquired pneumonia	28/32 (88%)
Hospital-acquired pneumonia	32/39 (82%)
Ventilator-acquired pneumonia	12/17 (71%)
Abdominal	34/36 (91%)
Secondary peritonitis	21/24 (88%)
Primary peritonitis	2/2 (100%)
Intra-abdominal abscess	3/5 (70%)
Bloodstream	30/34 (94%)
Line infection	22/26 (85%)
Primary bloodstream infection	4/5 (80%)
Central nervous system	10/11 (91%)
Secondary meningitis	5/7 (79%)
Primary meningitis	4/4 (100%)

## Conclusion

In this study, overall interobserver agreement of classifying infections using CDC criteria was excellent, whereas an exact match on all aspects of the diagnosis between independent observers was limited for some infections.

